# Cancer stage at diagnosis: Comparison of insurance status in SEER to the Department of Defense Cancer Registry

**DOI:** 10.1002/cam4.6655

**Published:** 2023-10-30

**Authors:** James T. Flanary, Jie Lin, Craig D. Shriver, Kangmin Zhu

**Affiliations:** ^1^ Department of Surgery Walter Reed National Military Medical Center Bethesda Maryland USA; ^2^ Department of Surgery, Murtha Cancer Center Research Program Uniformed Services University of the Health Sciences Bethesda Maryland USA; ^3^ The Henry M. Jackson Foundation for the Advancement of Military Medicine, Inc. Bethesda Maryland USA; ^4^ Department of Preventive Medicine and Biostatistics Uniformed Services University of the Health Sciences Bethesda Maryland USA

**Keywords:** early detection of cancer, military personnel/statistics and numerical data, neoplasm staging, SEER program, United States/epidemiology

## Abstract

**Background:**

Military individuals, retirees, and their families have free care or minimal out‐of‐pocket costs in the US military health system (MHS). In contrast, out‐of‐pocket costs in the US general population vary substantially. This study compared cancer patients with various insurance types in the general population to those in the MHS in cancer stage at diagnosis.

**Methods:**

Patients were identified from the US Department of Defense's (DoD) Automated Central Tumor Registry (ACTUR) and the National Cancer Institute's Surveillance, Epidemiology, and End Results (SEER) program. Tumor stage at diagnosis of breast, prostate, lung, and colon cancers during 2007–2013 was compared between ACTUR and SEER insurance categories of “insured,” “insured‐no specifics,” “any Medicaid,” and “uninsured,” A multivariable logistic regression analysis estimated the odds ratio (OR) of late stage (Stages III and IV) versus early stage (Stages I and II) cancers comparing SEER insurance status to ACTUR.

**Results:**

There were 18,440 eligible patients identified from ACTUR and 831,959 patients identified from SEER. For all cancer types, patients in the SEER‐insured/no specifics, Medicaid, and uninsured groups had significantly greater likelihood of late stage diagnosis compared to ACTUR patients. The adjusted ORs were greatest among uninsured and Medicaid patients. The SEER‐insured group also had a significantly higher odds of advanced stage disease than ACTUR patients for prostate cancer and lung cancer.

**Conclusion:**

Patients in the MHS with universal access to healthcare were diagnosed at an earlier stage than those in the general population. This difference was most evident compared to Medicaid and uninsured groups.

## 1 INTRODUCTION

Early detection of cancer results in decreased mortality,^1^, ^2^, ^3^ and in order for a malignancy to be detected and diagnosed, a person must have access to medical care. At present, the United States remains without a means in which to provide effective healthcare for its entire population.^4^, ^5^ Annual premiums for a typical employer sponsored plan were over $4000 in 2012 and have increased since.^6^ In the same year, approximately half of Americans were covered by an employer‐sponsored plan, 18% received coverage through Medicaid, and 15% were uninsured.^5^ The percent of uninsured had decreased to 9% in 2019 with commensurate increases in other categories.^5^ In the military health system (MHS), beneficiaries receive healthcare at no to minimal out‐of‐pocket costs. This includes primary care, cancer screening, diagnostic imaging, and cancer treatment. Beneficiaries include active duty members, retirees, and their family members.

Insurance status has been shown to affect cancer stage at diagnosis, with higher odds of advanced‐stage disease in patients with Medicaid or uninsured, compared to privately insured patients.^7^, ^8^ Expanding Medicaid through the Affordable Care Act (ACA) is associated with lower odds of metastatic disease at presentation.^9^ After health insurance expansion in Massachusetts, there was a decrease in the likelihood of presentation with advanced stage colorectal and breast cancers.^10^

The MHS provides universal health care to its beneficiaries. Our previous studies showed that MHS beneficiaries had earlier stages of colon, breast, and lung cancers compared to the population in the National Cancer Institute's Surveillance, Epidemiology, and End Results (SEER) registry.^11^, ^12^, ^13^ However, these studies did not subdivide the SEER population by insurance status category. Comparison of each insurance status to the MHS can offer more nuanced evidence of its association with stage at diagnosis. Using SEER and the US Department of Defense (DoD)'s Automated Central Tumor Registry (ACTUR), we examined the association of insurance status in SEER and the MHS with stage at diagnosis for lung, prostate, breast, and colon cancers.

## 2 METHODS

### 2.1 Data sources

This is a cross‐sectional study using data from two sources: the DoD ACTUR and the National Cancer Institute's SEER program. ACTUR was the DoD's cancer registry that tracks patients diagnosed and/or treated at military treatment facilities (MTFs). MTFs were required to report cancer diagnoses to ACTUR. Patients in ACTUR included active‐duty members and their family members, retirees and their family members, and Reserve and National Guard personnel who were temporarily activated. ACTUR complied with the uniform data standards set by the North American Association of Central Cancer Registries.^14^ The registry included data on demographics, tumor characteristics (stage, grade, tumor size, etc.), cancer treatment, and vital status of patients.

Patients from the US general population were identified from the SEER program database. The SEER program is a collection of cancer registries that collects standard data items on demographics, tumor characteristics, treatments, follow‐up, and other information.^15^ In this study, we used SEER 18 with catchments for the 18 SEER registries (Atlanta, Connecticut, Detroit, Hawaii, Iowa, New Mexico, San Francisco‐Oakland, Seattle‐Puget Sound, Utah, Los Angeles, San Jose‐Monterey, Rural Georgia, the Alaska Native, Greater California, Greater Georgia, Kentucky, Louisiana, and New Jersey). The SEER 18 covers about 28% US population.^16^

ACTUR data are non‐identifiable and SEER data are de‐identified for public use. The study was approved by the institutional review boards of Walter Reed National Military Medical Center and Uniformed Services University of the Health Sciences.

### 2.2 Study populations

The study population included those with a first cancer of invasive lung, prostate, breast, or colon, histologically diagnosed between January 1, 2007 and December 31, 2013 and who were 18 years or older at the time of diagnosis. These were the four most common cancers in terms of incidence and mortality,^17^ which also represent those in which cancer screening was available (breast, colon, and prostate cancers) and not available (lung cancer in the study period). This time period was selected because SEER included insurance data starting in 2007 and 2013 is the latest year of available ACTUR data. The International Classification of Diseases for Oncology, third edition codes were used to identify lung cancer (C34.0, C34.1, C34.2, C34.3, C34.8, C34.9), prostate (C61.9), breast cancer (C50.0, C50.1, C50.2, C50.3, C50.4, C50.5, C50.6, C50.8, C50.9), and colon cancer (C18.0, C18.2, C18.3, C18.4, C18.5, C18.6, C18.7, C18.8, C18.9).^18^ Given that over 95% of prostate and colon cancers belong to adenocarcinoma histology, only patients with adenocarcinoma were included for these cancers.^19^, ^20^ For lung and breast cancer, all histological types were included. Male breast cancer was excluded. Patients with multiple primary cancers were excluded.

### 2.3 Study variables

The study outcome was tumor stage at diagnosis. We defined tumor stage according to the American Joint Committee on Cancer's TNM system criteria including Stages I, II, III, and IV.^21^ Stages I and II were grouped as early stage and Stages III and IV were grouped as late stage. Other variables included age at diagnosis, gender (male, female), race (White, Black, Asian/Pacific Islander), ethnicity (Non‐Hispanic, Hispanic, unknown), year of diagnosis, and histology (lung and breast cancers). For breast cancer, histologic types were grouped into invasive ductal carcinoma, invasive lobular carcinoma, and other types. In addition, the combination of estrogen receptor status (ER) and progesterone receptor status (PR) was used to classify breast tumor as the following categories: ER+/PR+, ER−/PR+, ER+/PR−, or ER−/PR−. Given that human epidermal growth factor receptor 2 (HER2) data were not available for most patients in SEER and ACTUR during the study period, HER2 status was not included.^22^ For lung cancer, histologic types included both non‐small cell lung cancer and small cell lung cancer.

The variable of study was insurance status. All patients in ACTUR were MHS beneficiaries and were analyzed as a single group. The SEER insurance categories included “insured (private insurance, Medicare administered through a managed care plan, Medicare with private supplement),” “insured‐no specifics (Medicare NOS, insurance NOS),” “any Medicaid (Indian/Public health Service, Medicaid, Medicaid administered through a managed care plan, Medicare with Medicaid eligibility),” and “uninsured (no insurance, self‐pay)” as defined by SEER.^23^ There were 34,773 (3.9%) out of 885,172 patients in the SEER database who had unknown insurance status and were excluded from analysis.^23^

### 2.4 Statistical analysis

First, we conducted descriptive analysis comparing the distributions of demographic variables by insurance status. We then conducted multivariable unconditional logistic regression analysis to calculate odds ratios (ORs) of late stage (Stages III and IV) versus early stage (Stages I and II) diagnosis for each SEER insurance subgroup relative to ACTUR. This analysis was adjusted for age, sex (lung and colon cancer only), race, Hispanic ethnicity, and year of diagnosis. For breast cancer, the analysis was further adjusted for histologic type (invasive ductal carcinoma, invasive lobular carcinoma, and other types) and ER/PR status (ER+/PR+, ER−/PR+, ER+/PR−, or ER−/PR−). For lung cancer, the analysis was further adjusted for histology (small cell and non‐small cell).^19^, ^20^ The logistic analysis was further stratified by age and race. Age was grouped into <50, 50–64, and 65 or older. All statistical analyses were conducted using SAS software 9.4.0 (SAS Institute, Inc.).

## 3 RESULTS

A total of 850,399 patients were included in analysis, 18,440 of which were from ACTUR and 831,959 were from SEER. Tables 1 and 2 show demographic distributions by insurance status for each cancer site. For all cancer sites, the SEER‐insured/no specifics group contained a higher proportion of patients ages 65 or older than other groups. The SEER insured and SEER insured/no specifics groups had a higher percentage of White race and Hispanic ethnicity than other groups. In regard to histology and hormone receptor status of breast cancer, Medicaid and uninsured groups were less likely to have lobular type and more likely to have ER−/PR− tumors, compared to the other groups. For lung cancer, the Medicaid and no‐insurance groups had a higher proportion of small‐cell histology.

**TABLE 1:** Demographics and tumor characteristics of patients diagnosed with sex‐specific cancers (prostate and female breast cancer), during 2007–2013, in ACTUR and SEER registries.

	ACTUR	SEER‐insured	SEER‐insured/no specifics	SEER‐any Medicaid	Uninsured	*p*‐Value
	*N* (%)	*N* (%)	*N* (%)	*N* (%)	*N* (%)	
**Breast**						
Age group						
<50	2123 (34.5)	48,999 (23.8)	7262 (18.4)	10,108 (31.1)	2075 (38.0)	<0.0001
50–64	2605 (42.4)	80,632 (39.2)	13,003 (32.9)	13,954 (42.9)	2864 (52.4)	
65 or older	1420 (23.1)	76,167 (37.0)	19,281 (48.8)	8440 (26.0)	25 (9.6)	
Race						
White	4225 (68.7)	169,316 (82.3)	31,981 (80.9)	22,326 (68.7)	3760 (68.8)	<0.0001
Black	1172 (19.1)	19,323 (9.4)	4759 (12.0)	6631 (20.4)	1229 (22.5)	
Asian/Pacific Islander	751 (12.2)	17,159 (8.3)	2806 (7.1)	3545 (10.9)	475 (8.7)	
Hispanic ethnicity						
Non‐Hispanic	5254 (85.5)	187,879 (91.3)	35,907 (90.8)	24,338 (74.9)	4127 (75.5)	<0.0001
Hispanic	371 (6.0)	17,919 (8.7)	3639 (9.2)	8164 (25.1)	1337 (24.5)	
Unknown	523 (8.5)	0 (0.0)	0 (0.0)	0 (0.0)	0 (0.0)	
Histology						
Invasive ductal	4862 (79.1)	160,970 (78.2)	31,057 (78.5)	26,048 (80.1)	4353 (79.7)	<0.0001
Invasive lobular	501 (8.1)	18,698 (9.1)	3414 (8.6)	2257 (6.9)	372 (6.8)	
Other	785 (12.8)	26,130 (12.7)	5075 (12.8)	4197 (12.9)	739 (13.5)	
ER/PR status						
ER+/PR+	3956 (64.4)	11,125 (64.7)	2043 (64.5)	1840 (58.1)	313 (52.3)	<0.0001
ER+/PR−	670 (10.9)	1942 (11.3)	358 (11.3)	352 (11.1)	74 (12.4)	
ER−/PR+	67 (1.1)	200 (1.2)	41 (1.3)	61 (1.9)	9 (1.5)	
ER−/PR−	1201 (19.6)	3321 (19.3)	578 (18.2)	774 (24.4)	168 (28.1)	
Unknown	249 (4.1)	597 (3.5)	150 (4.7)	138 (4.4)	35 (5.8)	
**Prostate**						
Age group						
<50	634 (8.1)	7856 (3.8)	1130 (2.3)	471 (3.4)	262 (5.5)	<0.0001
50–64	4089 (56.2)	96,585 (47.1)	16,403 (33.9)	5552 (40.5)	3506 (73.9)	
65 or older	2553 (35.1)	100,827 (49.1)	30,853 (63.8)	7703 (56.1)	978 (20.6)	
Race						
White	4849 (66.6)	166,385 (81.1)	37,567 (77.6)	8336 (60.7)	3022 (63.7)	<0.0001
Black	2058 (28.3)	29,435 (14.3)	8645 (17.9)	3670 (26.7)	1506 (31.7)	
Asian/Pacific Islander	369 (5.1)	9448 (4.6)	2174 (4.5)	1720 (12.5)	218 (4.6)	
Hispanic ethnicity						
Non‐Hispanic	6177 (84.9)	188,987 (92.1)	44,146 (91.2)	10,255 (74.7)	3827 (80.6)	<0.0001
Hispanic	394 (5.4)	16,281 (7.9)	4240 (8.8)	3471 (25.3)	919 (19.4)	
Unknown	705 (9.7)	0 (0.0)	0 (0.0)	0 (0.0)	0 (0.0)	

Abbreviations: ACTUR, Automated Central Tumor Registry; SEER, Surveillance, Epidemiology, and End Results.

**TABLE 2:** Demographics and tumor characteristics of patients diagnosed with cancers among men and women (lung and colon cancer), during 2007–2013, in ACTUR and SEER registries.

	ACTUR	SEER‐insured	SEER‐insured/no specifics	SEER‐any Medicaid	SEER‐uninsured	*p*‐Value
	*N* (%)	*N* (%)	*N* (%)	*N* (%)	*N* (%)	%
**Colon**						
Age group						
<50	371 (20.4)	7123 (10.0)	1284 (6.9)	1697 (13.6)	1009 (26.0)	<0.0001
50–64	773 (42.5)	21,855 (30.6)	4432 (24.0)	4521 (36.3)	2521 (64.8)	
65 or older	674 (37.1)	42,479 (59.5)	12,791 (69.1)	6227 (50.0)	358 (9.2)	
Sex						
Male	1091 (60.0)	34,869 (48.8)	9380 (50.7)	5629 (45.2)	2153 (55.4)	<0.0001
Female	727 (40.0)	36,588 (51.2)	9127 (49.3)	6816 (54.8)	1735 (44.6)	
Race						
White	1297 (71.3)	58,566 (82.0)	14,337 (77.5)	8031 (64.5)	2590 (66.6)	<0.0001
Black	370 (20.4)	7748 (10.8)	2748 (14.9)	2602 (20.9)	988 (25.4)	
Asian/Pacific Islander	151 (8.3)	5143 (7.2)	1422 (7.7)	1812 (14.6)	310 (8.0)	
Hispanic ethnicity						
Non‐Hispanic	1573 (86.5)	65,142 (91.2)	16,751 (90.5)	10,061 (80.5)	3194 (82.2)	<0.0001
Hispanic	114 (6.3)	6315 (8.8)	1756 (9.5)	2429 (19.5)	694 (17.9)	
Unknown	131 (7.2)	0 (0.0)	0 (0.0)	0 (0.0)	0 (0.0)	
**Lung**						
Age group						
<50	190 (5.9)	4654 (4.3)	1148 (3.7)	2325 (9.7)	1092 (17.5)	<0.0001
50–64	1182 (37.0)	32,622 (30.0)	8281 (26.5)	11,148 (46.3)	4454 (71.4)	
65 or older	1826 (57.1)	7143 (65.7)	21,780 (69.8)	10,586 (44.0)	693 (11.0)	
Sex						
Male	1960 (61.3)	56,781 (52.2)	17,605 (56.4)	12,717 (52.9)	3674 (58.9)	<0.0001
Female	1238 (38.7)	51,938 (47.8)	13,604 (43.6)	11,342 (47.1)	2565 (41.1)	
Race						
White	2514 (78.6)	92,908 (85.5)	25,060 (80.3)	16,638 (69.2)	4616 (74.0)	<0.0001
Black	404 (12.6)	9595 (8.8)	4278 (13.7)	4882 (20.3)	1257 (20.2)	
Asian/Pacific Islander	280 (8.7)	6216 (5.7)	1871 (6.0)	2539 (10.6)	366 (5.9)	
Hispanic ethnicity						
Non‐Hispanic	2848 (89.1)	103,457 (95.2)	29,401 (94.2)	21,379 (88.9)	5683 (91.1)	<0.0001
Hispanic	119 (3.7)	5262 (4.8)	1808 (5.8)	2680 (11.1)	556 (8.9)	
Unknown	231 (7.2)	0 (0.0)	0 (0.0)	0 (0.0)	0 (0.0)	
Histology						
Non‐small cell	2785 (87.1)	91,363 (84.0)	25,908 (83.0)	15,530 (77.8)	5073 (81.3)	<0.0001
Small cell	413 (12.9)	17,356 (16.0)	5301 (17.0)	4429 (22.2)	1166 (18.7)	

Abbreviations: ACTUR, Automated Central Tumor Registry; SEER, Surveillance, Epidemiology, and End Results.

Tables 3, 4, 5, 6 showed that for each cancer site, the overall adjusted odds of being diagnosed with advanced stage disease were higher in the SEER‐insured/no specifics, Medicaid, and uninsured groups, compared to the ACTUR group. While there was no significant difference between the SEER‐insured group and ACTUR for breast cancer (Table 3), the SEER‐insured group had higher odds of late‐stage diagnosis for prostate cancer (OR = 1.16, 95% CI = 1.08–1.25; Table 4). No significant difference between the SEER‐insured group and ACTUR was observed for colon cancer (Table 5). However, for lung cancer, similar to prostate cancer, the SEER‐insured group was more likely to be diagnosed with a late tumor stage relative to ACTUR (OR = 1.72, 95% CI = 1.61–1.88; Table 6).

**TABLE 3:** Overall and stratified ORs of late stage at diagnosis comparing ACTUR with SEER insurance status among breast cancer patients diagnosed during 2007–2013.

	ACTUR	SEER‐ins	SEER‐ins/no specifics	SEER‐Medicaid	SEER‐uninsured
**Breast**					
Overall					
Stage I–II	5165	174,604	32,137	23,316	3646
Stage III–IV	983	32,137	7409	9186	1818
Adj. OR^a^ (95% CI)	1 (ref)	1.02 (0.94–1.09)	1.32 (1.22–1.42)	2.09 (1.93–2.25)	2.55 (2.33–2.80)
Age					
<50					
Stage I–II	1706	40,225	5810	6877	1706
Stage III–IV	417	8774	1452	3231	417
Adj. OR^a^ (95% CI)	1 (ref)	1.01 (0.90–1.14)	1.15 (1.01–1.31)	1.98 (1.78–2.24)	1.95 (1.68–2.26)
50–64					
Stage I–II	2223	68,833	10,678	9853	1860
Stage III–IV	382	11,799	2325	4101	1004
Adj. OR^a^ (95% CI)	1 (ref)	1.04 (0.93–1.17)	1.31 (1.16–1.49)	2.43 (2.14–2.74)	3.09 (2.69–3.55)
65+					
Stage I–II	1236	65,546	15,649	6586	373
Stage III–IV	184	10,621	3632	1854	152
Adj. OR^a^ (95% CI)	1 (ref)	1.07 (0.91–1.26)	1.50 (1.27–1.78)	1.79 (1.51–2.12)	2.67 (2.08–3.44)
Race					
White					
Stage I–II	3587	144,332	26,193	16,178	2573
Stage III–IV	638	24,984	5788	6148	1187
Adj. OR^a^ (95% CI)	1 (ref)	1.00 (0.92–1.10)	1.30 (1.19–1.43)	2.09 (1.89–2.29)	2.46 (2.20–2.76)
Black					
Stage I–II	932	15,408	3594	4458	734
Stage III–IV	240	3915	1165	2173	495
Adj. OR^a^ (95% CI)	1 (ref)	1.09 (0.93–1.28)	1.46 (1.23–1.72)	2.06 (1.76–2.42)	2.70 (2.23–3.26)
Asian/Pacific Islander					
Stage I–II	646	14,864	2350	2680	339
Stage III–IV	105	2295	456	865	136
Adj. OR^a^ (95% CI)	1 (ref)	1.01 (0.81–1.25)	1.37 (1.08–1.73)	2.19 (1.74–2.75)	2.49 (1.85–3.35)

Abbreviations: ACTUR, Automated Central Tumor Registry; CI, confidence interval; OR, odds ratio; SEER, Surveillance, Epidemiology, and End Results.

^a^ ORs were estimated from a multivariable logistic regression model. OR was further adjusted for age (as continuous variable), sex, race, Hispanic ethnicity, year of diagnosis, and histology. In stratified analysis, the stratifying variable itself was not adjusted.

**TABLE 4:** Overall and stratified ORs of late stage at diagnosis comparing ACTUR with SEER insurance status among prostate cancer patients diagnosed during 2007–2013.

	ACTUR	SEER‐ins	SEER‐ins/no specifics	SEER‐Medicaid	SEER‐uninsured
**Prostate**					
Overall					
Stage I–II	6373	175,008	41,134	10,782	3570
Stage III–IV	903	30,260	7252	2944	1176
Adj. OR^a^ (95% CI)	1 (ref)	1.16 (1.08–1.25)	1.19 (1.10–1.29)	1.81 (1.66–1.97)	2.20 (1.99–2.43)
Age					
<50					
Stage I–II	581	6767	952	342	184
Stage III–IV	53	1089	178	129	78
Adj. OR^a^ (95% CI)	1 (ref)	1.51 (1.12–2.02)	1.76 (1.27–2.45)	3.70 (2.61–5.26)	4.10 (2.77–6.06)
50–64					
Stage I–II	3599	81,873	13,858	4159	2624
Stage III–IV	490	14,712	2545	1393	882
Adj. OR^a^ (95% CI)	1 (ref)	1.27 (1.14–1.40)	1.32 (1.18–1.47)	2.36 (2.10–2.65)	2.39 (2.11–2.70)
65+					
Stage I–II	2193	86,368	26,324	6281	762
Stage III–IV	360	14,459	4529	1422	216
Adj. OR^a^ (95% CI)	1 (ref)	0.97 (0.86–1.09)	0.99 (0.88–1.12)	1.27 (1.12–1.45)	1.62 (1.34–1.96)
Race					
White					
Stage I–II	4228	141,583	31,931	6458	2247
Stage III–IV	621	24,804	5636	1878	775
Adj. OR^a^ (95% CI)	1 (ref)	1.15 (1.05–1.26)	1.16 (1.06–1.28)	1.86 (1.68–2.06)	2.19 (1.95–2.48)
Black					
Stage I–II	1837	25,567	7444	2919	1167
Stage III–IV	221	3868	1201	751	339
Adj. OR^a^ (95% CI)	1 (ref)	1.21 (1.04–1.40)	1.29 (1.10–1.51)	2.04 (1.73–2.41)	2.31 (1.91–2.79)
Asian/Pacific Islander					
Stage I–II	308	7858	1759	1405	156
Stage III–IV	61	1590	415	315	62
Adj. OR^a^ (95% CI)	1 (ref)	1.01 (0.76–1.35)	1.21 (0.89–1.65)	1.17 (0.86–1.60)	1.97 (1.31–2.97)

Abbreviations: ACTUR, Automated Central Tumor Registry; CI, confidence interval; OR, odds ratio; SEER, Surveillance, Epidemiology, and End Results.

^a^ ORs were estimated from a multivariable logistic regression model. OR was further adjusted for age (as continuous variable), sex, race, Hispanic ethnicity, year of diagnosis and histology. In stratified analysis, the stratifying variable itself was not adjusted.

**TABLE 5:** Overall and stratified ORs of late stage at diagnosis comparing ACTUR with SEER insurance status among colon cancer patients diagnosed during 2007–2013.

	ACTUR	SEER‐ins	SEER‐ins/no specifics	SEER‐Medicaid	SEER‐uninsured
**Colon**					
Overall					
Stage I–II	925	37,314	9348	5549	1500
Stage III–IV	893	34,143	9159	6896	2388
Adj. OR^a^ (95% CI)	1 (ref)	1.04 (0.94–1.27)	1.14 (1.03–1.26)	1.31 (1.18–1.45)	1.47 (1.31–1.65)
Sex					
Male					
Stage I–II	554	18,175	4683	2449	817
Stage III–IV	537	16,694	4697	3180	1336
Adj. OR^a^ (95% CI)	1 (ref)	0.99 (0.87–1.12)	1.10 (0.96–1.25)	1.30 (1.14–1.49)	1.47 (1.27–1.72)
Female					
Stage I–II	371	19,139	4665	3100	683
Stage III–IV	356	17,449	4462	3716	1052
Adj. OR^a^ (95% CI)	1 (ref)	1.11 (0.95–1.29)	1.20 (1.02–1.40)	1.35 (1.15–1.59)	1.48 (1.24–1.78)
Age					
<50					
Stage I–II	139	2820	487	535	339
Stage III–IV	232	4303	797	1162	670
Adj. OR^a^ (95% CI)	1 (ref)	0.93 (0.74–1.16)	1.01 (0.79–1.30)	1.30 (1.02–1.65)	1.19 (0.92–1.54)
50–64					
Stage I–II	406	10,956	2111	1779	1004
Stage III–IV	367	10,899	2321	2742	1517
Adj. OR^a^ (95% CI)	1 (ref)	1.09 (0.94–1.27)	1.21 (1.03–1.42)	1.67 (1.43–1.96)	1.64 (1.39–1.95)
65+					
Stage I–II	380	23,538	6750	3235	157
Stage III–IV	294	18,941	6041	2992	201
Adj. OR^a^ (95% CI)	1 (ref)	1.04 (0.89–1.22)	1.13 (0.96–1.33)	1.13 (0.96–1.33)	1.54 (1.18–2.00)
Race					
White					
Stage I–II	688	31,216	7402	3654	1023
Stage III–IV	609	27,350	6935	4377	1567
Adj. OR^a^ (95% CI)	1 (ref)	1.05 (0.94–1.18)	1.15 (1.02–1.30)	1.36 (1.20–1.53)	1.52 (1.33–1.75)
Black					
Stage I–II	171	3594	1265	1082	374
Stage III–IV	199	4154	1483	1520	614
Adj. OR^a^ (95% CI)	1 (ref)	1.06 (0.85–1.32)	1.14 (0.91–1.43)	1.27 (1.02–1.60)	1.34 (1.04–1.72)
Asian/Pacific Islander					
Stage I–II	66	2504	681	813	103
Stage III–IV	85	2639	741	999	207
Adj. OR^a^ (95% CI)	1 (ref)	0.87 (0.62–1.22)	0.99 (0.70–1.41)	1.10 (0.78–1.56)	1.49 (0.99–2.25)

Abbreviations: ACTUR, Automated Central Tumor Registry; CI, confidence interval; OR, odds ratio; SEER, Surveillance, Epidemiology, and End Results.

^a^ ORs were estimated from a multivariable logistic regression model. OR was further adjusted for age (as continuous variable), sex, race, Hispanic ethnicity, year of diagnosis and histology. In stratified analysis, the stratifying variable itself was not adjusted.

**TABLE 6:** Overall and stratified ORs of late stage at diagnosis comparing ACTUR with SEER insurance status among lung cancer patients diagnosed during 2007–2013.

	ACTUR	SEER‐ins	SEER‐ins/no specifics	SEER‐Medicaid	SEER‐uninsured
**Lung**					
Overall					
Stage I–II	1204	28,781	7555	4814	839
Stage III–IV	1989	79,938	23,654	19,245	5400
Adj. OR^a^ (95% CI)	1 (ref)	1.74 (1.61–1.88)	1.91 (1.76–2.07)	2.22 (2.04–2.41)	3.32 (2.98–3.69)
Sex					
Male					
Stage I–II	718	13,894	3968	2244	437
Stage III–IV	1242	42,887	13,637	10,473	3237
Adj. OR^a^ (95% CI)	1 (ref)	1.74 (1.58–1.93)	1.89 (1.71–2.10)	2.28 (2.04–2.54)	3.34 (2.90–3.85)
Female					
Stage I–II	491	14,887	3587	2570	402
Stage III–IV	747	37,051	10,017	8772	2163
Adj. OR^a^ (95% CI)	1 (ref)	1.73 (1.53–1.95)	1.91 (1.68–2.17)	2.13 (1.87–2.42)	3.21 (2.73–3.78)
Age					
<50					
Stage I–II	76	937	211	316	155
Stage III–IV	114	3717	937	2009	937
Adj. OR^a^ (95% CI)	1 (ref)	2.52 (1.83–3.45)	2.65 (1.88–3.73)	3.82 (2.74–5.31)	3.67 (2.58–5.22)
50–64					
Stage I–II	405	7490	1816	1898	560
Stage III–IV	777	25,132	6465	9250	3894
Adj. OR^a^ (95% CI)	1 (ref)	1.78 (1.56–2.03)	1.81 (1.58–2.08)	2.40 (2.09–2.75)	3.46 (2.96–4.05)
65+					
Stage I–II	728	20,354	5528	2600	124
Stage III–IV	1098	51,089	16,252	7986	569
Adj. OR^a^ (95% CI)	1 (ref)	1.65 (1.49–1.83)	1.88 (1.69–2.09)	1.90 (1.70–2.12)	2.80 (2.25–3.50)
Race					
White					
Stage I–II	949	25,028	6203	3390	627
Stage III–IV	1565	67,880	18,857	13,248	3989
Adj. OR^a^ (95% CI)	1 (ref)	1.67 (1.53–1.83)	1.83 (1.67–2.01)	2.13 (1.94–2.34)	3.26 (2.89–3.68)
Black					
Stage I–II	139	2167	906	895	166
Stage III–IV	265	7428	3372	3987	1091
Adj. OR^a^ (95% CI)	1 (ref)	1.75 (1.40–2.18)	1.88 (1.49–2.37)	2.15 (1.71–2.71)	2.93 (2.23–3.86)
Asian/Pacific Islander					
Stage I–II	121	1586	446	529	46
Stage III–IV	159	4630	1425	2010	320
Adj. OR^a^ (95% CI)	1 (ref)	2.45 (1.89–3.16)	2.80 (2.13–3.66)	3.34 (2.56–4.36)	5.09 (3.42–7.58)

Abbreviations: ACTUR, Automated Central Tumor Registry; CI, confidence interval; OR, odds ratio; SEER, Surveillance, Epidemiology, and End Results.

^a^ ORs were estimated from a multivariable logistic regression model. OR was further adjusted for age (as continuous variable), sex, race, Hispanic ethnicity, year of diagnosis and histology. In stratified analysis, the stratifying variable itself was not adjusted.

In breast cancer, stratified analysis showed that the Medicaid group had higher odds of late‐stage diagnosis, relative to ACTUR, in the 50–64 age group (OR = 2.43, 95% CI = 2.14–2.74) than the group aged 65 or older (OR = 1.79, 95% CI = 1.51–2.12; Table 3). Similar results were observed for colon cancer (OR = 1.67, 95% CI = 1.43–1.96 for ages 50–64, and OR = 1.13, 95% CI = 0.96–1.33 for ages 65 or older, respectively; Table 5). In prostate cancer, each SEER group had higher odds of late‐stage diagnosis relative to ACTUR in the <50 age group than the group 65 years or older (Table 4). In lung cancer, the Medicaid group had a higher OR of late stage diagnosis in the <50 age group (OR = 3.82, 95% CI = 2.74–5.31) than the group 65 years or older (OR = 1.90, 95% CI = 1.70–2.12; Table 6). Otherwise, results stratified by age groups were similar to the overall adjusted results for each cancer type.

The results stratified by racial groups were similar to the overall results for each cancer type. However, in lung cancer, the SEER‐insured, SEER‐insured/no specifics, and SEER‐Medicaid groups had higher odds of late stage diagnosis, relative to ACTUR, for Asian/Pacific Islanders than Whites (Table 6). When stratified by sex, results were similar to the overall adjusted results for both cancer types in which this analysis was performed (Tables 5 and 6). In stratified analysis by histology (performed in lung cancer and breast cancer) and ER/PR status (breast cancer), results were similar to the overall adjusted results (Results not shown).

## 4 DISCUSSION

Our results show a later stage at diagnosis for breast, prostate, colon, and lung cancers in Medicaid and uninsured groups compared to the MHS, across age groups. We also found a later stage at diagnosis for prostate and lung cancer for privately insured groups compared to the MHS. The Medicaid and uninsured groups had the highest odds of late stage diagnosis. There are several factors to consider in interpreting these findings, including the role of costs, access to care, cancer screening, and characteristics of the populations.

The most striking system‐related difference between ACTUR and SEER and between SEER insurance groups is the costs and fee schedules. Costs for uninsured patients are often prohibitive, which likely explains why odds of later stage diagnosis were highest among uninsured patients. Costs for privately insured patients are highly variable. Some have very low out‐of‐pocket costs, whereas some face higher costs through high‐deductible health plans or other plans. On average, out‐of‐pocket costs for privately insured beneficiaries were at least $4000 more when compared to those in the MHS.^24^, ^25^ Out‐of‐pocket costs thus may contribute to the differences observed in stage at diagnosis between the MHS and SEER‐insured groups. Later diagnosis among Medicaid patients cannot be explained by cost sharing, given the very low out‐of‐pocket costs for these patients. However, uninsured patients are often enrolled in and classified under Medicaid shortly after a new cancer diagnosis.^26^ This may also contribute to later stage diagnosis in this group.

Access to care may also provide some explanation for our findings. Medicaid patients face more barriers in finding physicians who accept their insurance and are more likely to have delays in care due to cost,^27^ all of which could possibly lead to a delay in diagnosis. Delays in time to appointments could also be a factor affecting stage at diagnosis between ACTUR and SEER‐insured groups, especially in the case of lung cancer; waiting times for appointments in the MHS are shorter than that reported in a separate survey among civilian physicians.^28^, ^29^ Lung cancer has wide variation in time to diagnosis, often lasting several months.^30^ Factors such as time to initial presentation, time to specialist referral, time to diagnostic testing, and time to definitive treatment, all contribute. Logistical barriers in the health system, such as waiting times or administrative obstacles to obtaining care, combined with out‐of‐pocket costs, could contribute to later diagnosis.

Screening is an important path to diagnosis for breast, colon, and prostate cancers and may be one explanation for our findings. Higher utilization of screening services in the ACTUR population may contribute to earlier stage at diagnosis. While breast cancer screening rates may be similar among patients with military insurance and private insurance, screening rates are lower among patients with Medicaid and especially for uninsured patients.^31^, ^32^, ^33^ For colon cancer, reported rates of screening are higher among patients with military insurance, whereas screening is less common among patients with Medicaid and least common among uninsured patients.^34^ Findings for prostate cancer may also be associated with cancer screening. Early stage prostate cancer, which is almost always detected via screening, had a higher incidence in the military population.^35^ There is also a higher rate of prostate cancer screening in the military compared to the general population during this time.^36^ However, lung cancer reflects a much different path to diagnosis than other presented cancers. There was no standardized lung cancer screening during the interval of this study.

Stratified analysis revealed larger ORs for younger groups (50–64 years and <50 years old) in some cancers. Forgoing earlier medical visits for a symptom or sign due to cost may be more prominent in younger patients than older ones. This may be particularly emphasized in lung cancer, in which the significance of suggestive symptoms is difficult to interpret and often initially presumed to be benign. It is also unknown whether smoking rates and availability or frequency of lung imaging in the SEER groups differed from the MHS population. The less prominent differences between ACTUR and SEER in the age 65 or older group may reflect converging screening practices at this age.

Notably, the data in our study are prior to many of the policies instituted by the ACA, such as implementation of the exchanges and elimination of cost‐sharing for preventive services.^37^ Cancer screening was already covered without any out‐of‐pocket costs for many patients prior to the ACA.^38^ The ACA eliminated out‐of‐pocket costs for cancer screening for those not previously covered.^39^ Elimination of cost‐sharing may increase use of these services and has been shown to have a small but detectable effect on detecting earlier stage cancer.^39^ The expanded screening offered through the ACA could potentially narrow the differences in stage at diagnosis between the MHS and the general population. However, this effect may be modest since many differences remain in the structure of military insurance compared to private insurance and Medicaid, especially in regard to out‐of‐pocket costs. Thus, our findings likely remain relevant in today's insurance landscape.

There are several limitations of the study. The active‐duty population in the MHS may represent a healthier cohort relative to the US population that also receives more frequent health screenings as part of military readiness. However, these patients comprise a small portion of patients with cancer in the ACTUR database. Among patients in ACTUR, the percentage who were active duty was 6.3% for breast cancer, 8.3% for prostate cancer, 10.5% for colon cancer, and 2.5% for lung cancer. The majority are family members, retirees, and their family members, whose overall health are more similar to the general population. Additionally, patients aged 65 or older in the MHS could also be included in SEER because of enrollment in Medicare. Nevertheless, this misclassification might only dilute the true difference between the MHS and the SEER‐insured group. Another limitation is the lack of data with respect to socioeconomic status and health behaviors in SEER and ACTUR. For example, patients living in residentially segregated neighborhoods may be at elevated cancer risk^40^ and may also be more likely to have Medicaid insurance or no insurance. An additional limitation is that access to care, use of preventive services, and screening may vary with geography across the US, and thus their effects could not be evaluated.

## 5 CONCLUSION

The presented findings provide evidence that patients in the MHS had diagnosis of common cancers at an earlier stage, compared to various insurance types in the general US population. This difference was largest compared to the uninsured and Medicaid groups, while it was minimal for the SEER‐insured groups. The effects are most accentuated in lung cancer. Our analysis highlights the association between insurance status and stage at cancer diagnosis.

## AUTHOR CONTRIBUTIONS

**James T. Flanary:** Conceptualization (equal); investigation (equal); methodology (supporting); visualization (equal); writing – original draft (lead); writing – review and editing (equal). **Jie Lin:** Conceptualization (equal); formal analysis (lead); investigation (equal); methodology (equal); visualization (equal); writing – review and editing (equal). **Craig D. Shriver:** Conceptualization (equal); funding acquisition (lead). **Kangmin Zhu:** Conceptualization (equal); investigation (equal); methodology (equal); visualization (equal); writing – review and editing (lead).

## FUNDING INFORMATION

This project was supported by the Murtha Cancer Center Research Program (MCCRP) of the Department of Surgery, Uniformed Services University of the Health Sciences (USUHS) under the auspices of the Henry M. Jackson Foundation for the Advancement of Military Medicine (HJF, Inc.).

## CONFLICT OF INTEREST STATEMENT

The authors have no conflict of interest to declare.

## DISCLAIMER

The contents of this manuscript are the sole responsibility of the authors and do not necessarily reflect the views, assertions, opinions, or policies of the USUHS, the Henry M. Jackson Foundation for the Advancement of Military Medicine Inc., the Department of Defense, or the Departments of the Army, Navy, or Air Force. Mention of trade names, commercial products, or organizations does not imply endorsement by the US government.

## Data Availability

The data that support the findings of this study come from two sources. A request for ACTUR data can be made to the Joint Pathology Center, in Silver Spring, MD. Data from SEER are publicly available.
